# Decreased IL-17 during treatment of sputum smear-positive pulmonary tuberculosis due to increased regulatory T cells and IL-10

**DOI:** 10.1186/s12967-016-0909-6

**Published:** 2016-06-16

**Authors:** Lichen Xu, Guangying Cui, Hongyu Jia, Yunan Zhu, Yulong Ding, Jianing Chen, Chong Lu, Ping Ye, Hainv Gao, Lanjuan Li, Weihang Ma, Jianxin Lyu, Hongyan Diao

**Affiliations:** The Key Laboratory of Laboratory Medicine, Ministry of Education of China, Zhejiang Provincial Key Laboratory of Medical Genetics, Wenzhou Medical University School of Laboratory Medicine and Life Sciences, Wenzhou, 325035 Zhejiang China; State Key Laboratory for Diagnosis and Treatment of Infectious Diseases, Collaborative Innovation Center for Diagnosis and Treatment of Infectious Diseases, The First Affiliated Hospital, School of Medicine, Zhejiang University, Hangzhou, 310003 Zhejiang China; Department of Hematology, The 3rd People’s Hospital Zhengzhou, Zhengzhou, 450001 Henan China

**Keywords:** IL-17, IL-10, Regulatory T cells, Pulmonary tuberculosis

## Abstract

**Background:**

Tuberculosis (TB) remains a major public health concern worldwide. Previous studies have demonstrated that IL-17 plays an important role in initial immune response and is involved in both immune-mediated protection and pathology following infection with *Mycobacterium tuberculosis* (MTB). However, the alterations and regulation of plasma IL-17 level during TB treatment remain unclear. Moreover, the cell type responsible for the production of IL-17 in TB patients requires further study.

**Methods:**

A total of 20 acid-fast bacilli smear-positive (AFB-positive) pulmonary TB patients and 20 age- and gender-matched healthy volunteers were included in our study. Blood samples were collected in heparinized tubes at the time of diagnosis (AFB-positive group) and 3 weeks after the initiation of therapy, when the sputum smear conversion (AFB-negative group) occurred, followed by symptomatic improvement. IL-17 levels and IL-17-producing cells in PBMCs were detected. Lymphocyte populations in the peripheral blood between the AFB-positive and AFB-negative groups were compared by flow-cytometry. A549 cells, a cell line of alveolar epithelial cells, were applied to determine the extent of the pathological damage mediated by IL-17 following MTB infection. Recombinant human IL-10 was used to investigate the regulation of IL-17 expression after sputum smear conversion in AFB-positive pulmonary TB patients.

**Results:**

Plasma IL-17 level were elevated in patients with sputum AFB-positive pulmonary TB, but substantially decreased after TB treatment and smear conversion. Our data indicate that NKT-like cells might be the main source of IL-17, in addition to conventional T cells in AFB-positive pulmonary TB patients. The secretion of IL-17 may be suppressed by regulatory T (Treg) cells and IL-10 during TB treatment. Moreover, the IL-17 levels were positively correlated to both the C-reactive protein and erythrocyte sedimentation rate. Therefore, IL-17 was capable of alveolar epithelial cell damage following MTB infection.

**Conclusion:**

The increase in the frequency of Treg cells and IL-10 levels was associated with a decrease in IL-17 in patients receiving TB treatment. Thus, IL-10 and Tregs may function to inhibit immune-mediated pathology in TB patients.

## Background

Tuberculosis (TB) is a communicable respiratory disease caused by infection with *Mycobacterium tuberculosis* (MTB), and ranks as the second leading cause of death from the infectious diseases worldwide [1]. In 2013, there were 9 million new cases of TB diagnosed and 1.5 million deaths due to the disease [1]. Adaptive immune responses mediated by CD4^+^ T cells and CD8^+^ T cells, and T helper (Th) 1 cytokines characterized by interferon (IFN)-γ production are associated with a good prognosis and play an important role in countering the progression of MTB infection [2–4]. However, Th1 cells (primarily CD4^+^ cells producing IFN-γ) alone are not capable of controlling the infection [3, 5] and other factors, including Th2 cells, Th17 cells and regulatory T cells (Treg cells), are also involved in the progression of MTB infection.

Interleukin (IL)-17, also known as IL-17A, is a number of the IL-17 family which range from A to F [6, 7]. However, IL-17 is of particular importance as it is the cytokine primarily secreted by Th17 cells [6, 7]. IL-17 production can be efficiently induced from naive CD4^+^ T cell by the IL-23 or IL-6, independently of TGF-β [8]. Recent studies have shown that IL-17 plays an important role in the initial immune responses and is involved in both immune protection and immune pathology in MTB infection [2, 9, 10]. The Th17-response is also considered to be the leading mechanism of protection of bronchoalveolar tract and its barrier maintenance [11].

IL-17 producing CD4^+^ T cells, activated in response to vaccination, has been shown to inhibit bacterial growth in the lung after MTB infection, as well as promote the production of chemokines that recruit and activate neutrophils and IFN-γ producing CD4^+^ T cells [12–15]. Moreover, IL-17 is essential for the vaccine-induced protection against MTB infection by inducing the localization of the proinflammatory cytokine producing C-X-C motif chemokine receptor 5-positive (CXCR5^+^) T cells, thereby promoting early macrophage activation and the control of MTB [16]. In contrast, other studies demonstrated that IL-17 played an essential role in granuloma formation in the lung [17], and was involved in the pathological damage mediated by the initial neutrophil recruitment following MTB infection [18]. To limit this pathological damage, a serial of immune regulatory factors are in place, including regulatory T (Treg) cells and the production of the anti-inflammatory cytokine IL-10 [19, 20]. IL-10 production by Tregs can effectively inhibit not only IFN-γ expression but also the ability of CD4^+^ T cells and CD8^+^ T cells to degranulate in response to MTB [19]. However, the dynamic changes and regulation of IL-17 during TB treatment remains unclear. Moreover, the cell type that is the primary source of IL-17 in TB patients has not been identified to date.

Here, we recruited patients with sputum acid-fast bacilli smear-positive (AFB-positive) pulmonary TB, and compared the changes of plasma cytokines before the initiation of anti-TB therapy and after the sputum smear conversion. We found that plasma IL-17 was elevated in the AFB-positive patients, but substantially decreased following TB treatment and smear conversion. Moreover, IL-17 levels were positively correlated to both the C-reactive protein (CRP) and erythrocyte sedimentation rate (ESR). IL-17 also aggravated alveolar epithelial cells damage following MTB infection. Our findings further indicate that NKT-like cells might also be the main source of IL-17, in addition to conventional T cells in TB patients. Moreover, the secretion of IL-17 may be regulated by Treg cells and IL-10 during anti-TB treatment.

## Methods

### Patients and associated procedures

A total of 20 AFB-positive pulmonary TB patients and 20 age- and gender-matched healthy volunteers were recruited at the First Affiliated Hospital, School of Medicine, Zhejiang University from June 2014 to November 2014. All TB patients were diagnosed according to China’s TB diagnosis standard. Any patients that were co-infected with other pathogens or had autoimmune diseases were excluded from this study. Written informed consent was obtained from all patients and healthy volunteers. The study protocol was approved by the Ethics committee of the First Affiliated Hospital, School of Medicine, Zhejiang University (reference number 2015-312). The clinical characteristics for all of the study participants are presented in Table 1.

**Table 1:** Clinical characteristics of patients with pulmonary tuberculosis

	HC (n = 20)	AFB-positive TB patients (n = 20)
Age (years)	45 ± 21.9 (22–76)	55 ± 19.8 (18–86)
Sex (m/f)	12/8	13/7
*Radiographic features*		
Infiltration	–	20/20 (100)
Cavitation	–	7/20(35)
Effusion	–	3/20 (15)
*Smear grading*		
4+	–	5/20 (25)
3+	–	6/20 (30)
2+	–	5/20 (25)
1+	–	4/20 (20)
Resistance	–	–
Treatment	–	HREZ^a^ 20/20 (100)

^a^HERZ, isoniazid, ethambutol, rifampicin, plus pyrazinamide. Categorical variable data are presented as positive/tested (%). Continuous variable data are shown as mean ± SD (range)

### Sample collection

Blood samples were collected in heparinized tubes at the time of diagnosis (AFB-positive group) and 3 weeks after the initiation of therapy when the sputum smear conversion (AFB-negative group) occurred, followed by a symptomatic improvement. CRP and ESR were determined. Plasma was separated from peripheral blood mononuclear cells (PBMCs) at 3000 rpmfor 5 min and was stored at −80 °C until use. PBMCs were isolated by density centrifugation on with Ficoll, according to the manufacturer’s instructions.

### Flow cytometric analysis of lymphocyte subtypes

The following monoclonal antibodies were used in the present study: FITC-anti-CD4/PE-anti-CD8/Percp-anti-CD3, FITC-anti-CD4, PE-anti-CD25, APC-anti-CD127, FITC-anti-CD3/PE-anti-CD56 APC-anti-CD3, FITC-anti-CD56 (BD Pharmingen, San Diego, CA, USA), PE-anti-IL-17A and PE-anti-IFN-γ (eBioscience, ST, USA). Cells were stained according to standard procedures by incubating the antibodies at 4 °C for 30 min. Intracellular cytokine staining was performed by fixing and permeabilizing the cells with the Cytofix/Cytoperm Fixation/Permeabilization Kit (BD Pharmingen, CA, USA) according to the manufacturer’s instructions. Flow cytometry was performed on the BD canto II and the data was analyzed using BD Diva software (BD, San Diego, CA, USA).

### Cell culture

A549 cells are an alveolar type-II epithelial cell line, that were cultured in DMEM (Gibco, CA, USA), supplemented with 100 units/mL penicillin, 100 μg/mL streptomycin, and 10 % fetal bovine serum (FBS) (Gibco, CA) at 37 °C, 5 % CO_2_. Cells were seeded in 96-well tissue culture plates, following 60–70 % adherence. 100 ng/mL recombinant IL-17A (rOPN, R&D, MN, USA) and 15 μg/mL pulmonary *Mycobacterium bovis* bacille Calmette–Guerin (BCG, Shanghai Institute of Biological Products Co., Ltd, China) were added respectively. Cells and supernatants were harvested at 24 h. The supernatants were used to determine the concentration of lactate dehydrogenase (LDH) according to the manufacturer’s instructions (Roche, Mannheim, Germany). An analysis of cellular apoptosis was carried out according to the manufacturer’s instructions (BD Pharmingen, San Diego, CA, USA).

PBMCs from health volunteers were cultured at 37 °C, 5 % CO_2_ in RPMI 1640 (Gibco, CA, USA), supplemented with 100 units/mL penicillin, 100 μg/mL streptomycin, and 10 % FBS. Cells were incubated with recombinant IL-10 (10 μg/mL, Biolegend, CA, USA) and BCG (15 μg/mL). Golgi inhibitor was added for the last 5 h of the incubation. Cells were harvested at 24 h, and intracellular cytokine analysis of IL-17A production was conducted according to the manufacturer’s instructions.

### Cytokines analysis

IL-17A, IL-6, IL-23 and IFN-γ were determined by the Luminex enzyme immunoassay (Luminex, TX, USA) according to the manufacturer’s protocol (Millipore, Boston, MA, USA).

### ELISPOT assay

According to the manufacturer’s instructions, the IL-17A and IFN-γ ELISPOT assays were performed using commercially available kits (eBiosciences, USA). A total of 2 × 10^6^ PBMCs were cultured with PMA (25 ng/mL) and ionomycin (1 ng/mL) in complete RPMI 1640 medium at 37 °C for 24 h. After stimulation, the positive cells were enumerated by an ImmunoSpot S5 Macro Analyzer (C.T.L., Shaker Heights, OH, USA), and expressed as the numbers of IL-17A and IFN-γ spot-forming units per well.

### ELISA

ELISA kit for IL-10 (eBioscience, San Diego, CA, USA) was used to determine the concentrations of IL-10 according to the manufacturers’ instructions.

### Statistical analyses

Data are presented by box plot or mean ± SD. The significance of differences between two groups was determined using a non-parametric test. A correlation analysis was performed using the Pearson’s correlation coefficient analysis. A p value of less than 0.05 was considered to be statistical significance. *p < 0.05. **p < 0.01, ***p < 0.001. All analyses were performed using SPSS software.

## Results

### IL-17 level increased in AFB-positive patients but decreased after smear conversion

**Fig. 1:**
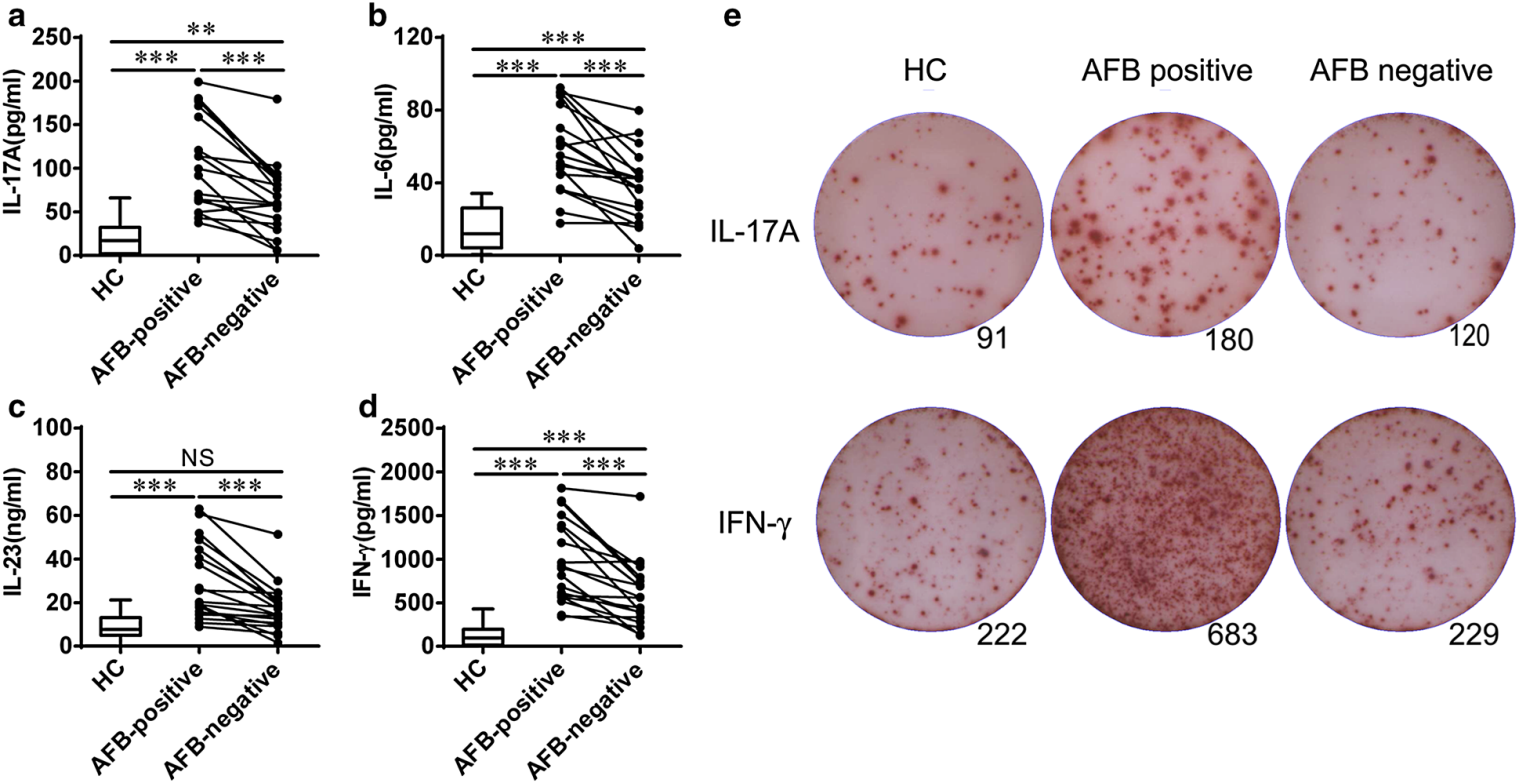
Elevated IL-17 levels in AFB-positive patients decreased after smear conversion. Plasma levels of **a** IL-17, **b** IL-6, **c** IL-23, and **d** IFN-γ in the AFB-positive group compared to the AFB-negative group were analyzed by ELISA. Data are presented by a *box plot*. **e** The expression of IL-17 and IFN- secreted by PBMCs from the AFB-positive group and AFB-negative group were determined by ELISPOT assays

Firstly, we detected plasma cytokines levels in patients with AFB-positive pulmonary TB and healthy controls. We found that plasma levels of IL-17, IL-6, IL-23 and IFN-γ were significantly elevated in the AFB-positive group compared to healthy controls (HC) (all p < 0.001) (Fig. 1a–d). After the effective treatment, the patients achieved AFB-negative status. We found that plasma levels of IL-17, IL-6, IL-23 and IFN-γ were remarkably decreased in the AFB-negative group in comparison with the smear-positive group (all p < 0.001) (Fig. 1a–d). However, the levels of IL-17, IL-6 and IFN-γ were also high in the AFB-negative group compared to healthy controls, except the level of IL-23 had no significant difference between the two groups.

To further verify the decrease of IL-17 level after smear conversion, ELISPOT assays were performed. We found that the numbers of IL-17- and IFN-γ-secreting cells were increased in the AFB-positive group compared to healthy controls (Fig. 1e). In contrast, the numbers of IL-17- and IFN-γ-secreting cells were decreased after smear conversion (Fig. 1e).

### Comparison of lymphocyte subgroups in the peripheral blood between the AFB-positive and AFB-negative groups

**Fig. 2:**
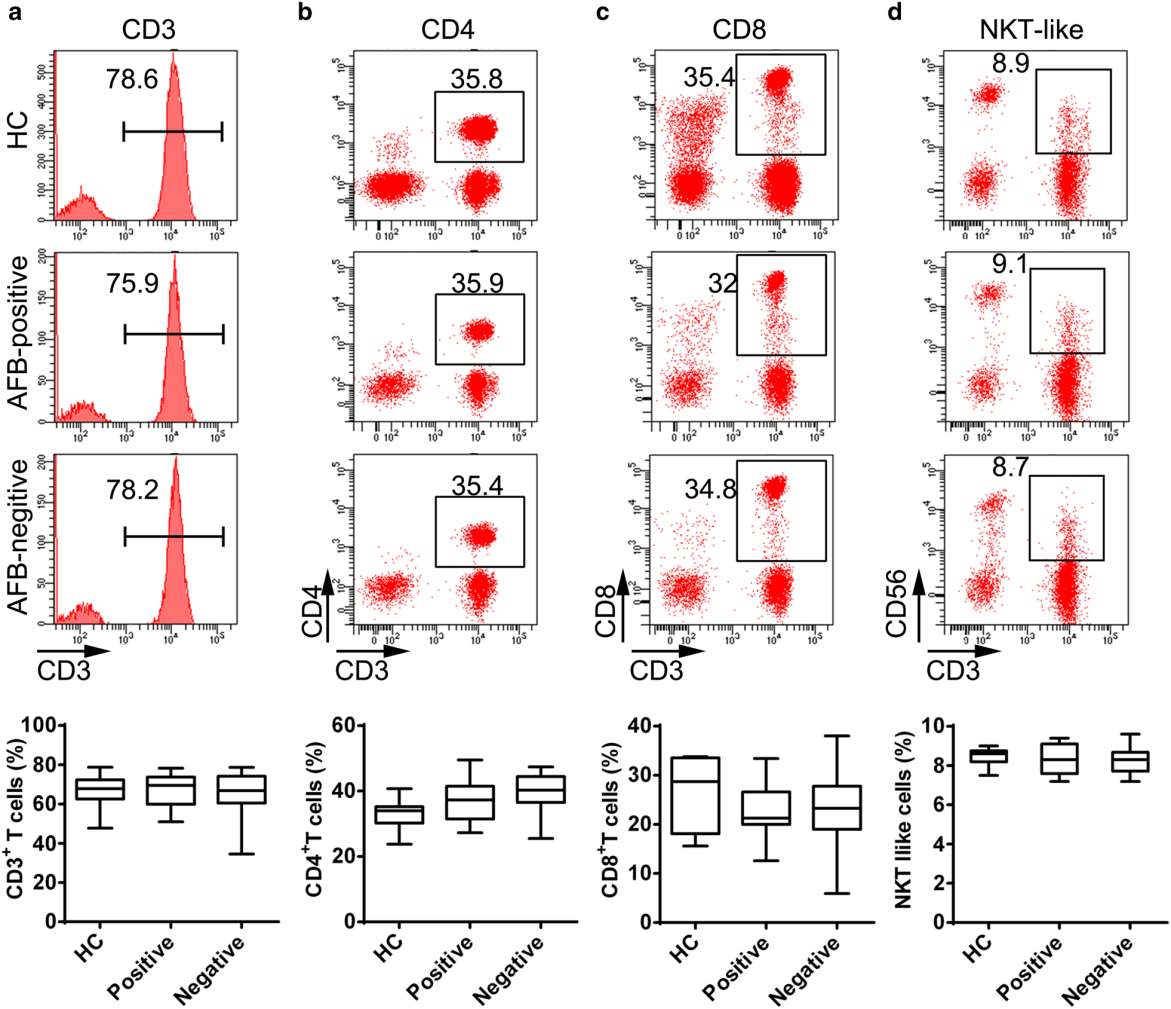
The frequencies of PBMC subsets from the AFB-positive group compared to the AFB-negative group. The distribution of **a** CD3^+^ cells, **b** CD4^+^ cells, **c** CD8^+^ cells, and **d** NKT-like cells out of the total PBMCs were analyzed, and the percentages of these cells were compared between the AFB-positive and AFB-negative groups by flow cytometry analysis

IL-17 is a pro-inflammatory cytokine mainly produced by Th17 cells [6, 7, 21], a subpopulation of T lymphocytes. Firstly, we observed the distribution of T lymphocyte populations. There were no significant differences in the frequencies of CD3^+^ T cells, CD4^+^ T cells and CD8^+^ T cells between the AFB-negative and AFB-positive groups (Fig. 2a–c).

Recently, it was reported that the activated NKT-like (CD3^+^CD56^+^) cells were capable of rapidly producing IL-17 [22]. Thus, we further observed the alterations of NKT-like cells and found that the percentage of NKT-like cells in the AFB-positive group was similar with that in the AFB-negative group (Fig. 2d).

### NKT-like cells produced significant amounts of IL-17

**Fig. 3:**
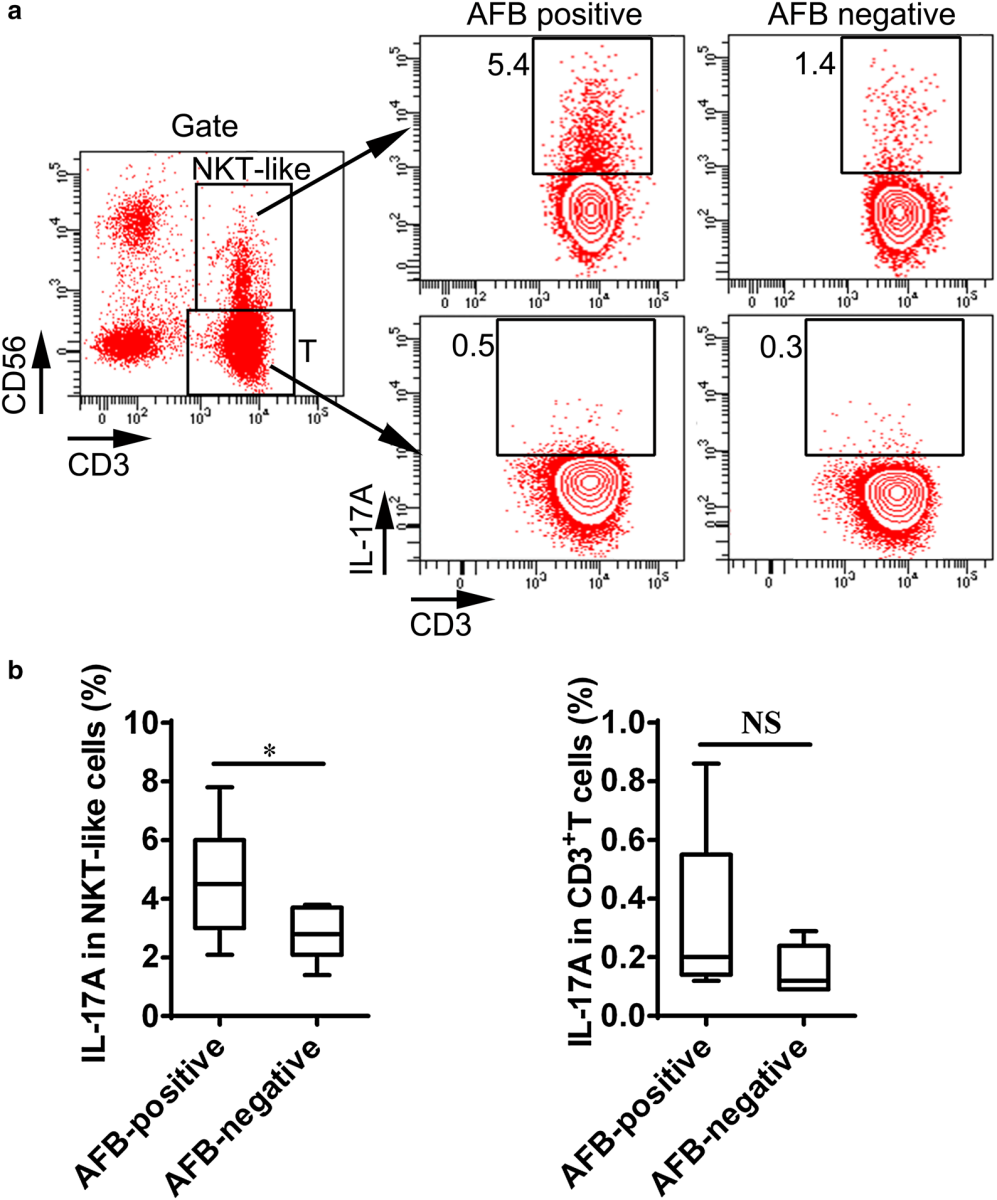
Comparison of the frequency of IL-17-producing cells in the peripheral blood between the AFB-positive and AFB-negative groups. **a** A *scatterplot* and **b** the frequencies of the IL-17-producing cell in the NKT-like cell and T cell populations from the AFB-positive group compared to the AFB-negative group. Data were presented by a *box plot*. *p < 0.05

To further investigate the source of IL-17 in TB patients, IL-17-producing cells in NKT-like cells (CD3^+^CD56^+^) and conventional T cells (CD3^+^CD56^−^) were detected. We found that the percentage of IL-17-producing cells in NKT-like cells was decreased in the AFB-negative group in comparison with the AFB-positive group (p = 0.0344) (Fig. 3a, b). The frequency of IL-17-producing cells in conventional T cells had a decreased trend but there was no statistical difference in the AFB-negative compared to AFB-positive groups (p = 0.1602) (Fig. 3a, b).

The decreased percentage of IL-17-producing cells in NKT-like was consistent with the low expression of plasma IL-17 level in TB patients after smear conversion, which suggested that NKT-like cells might also be an important source of IL-17, in addition to conventional T cells in TB patients.

### IL-17 level was correlated to both CRP and ESR

**Fig. 4:**
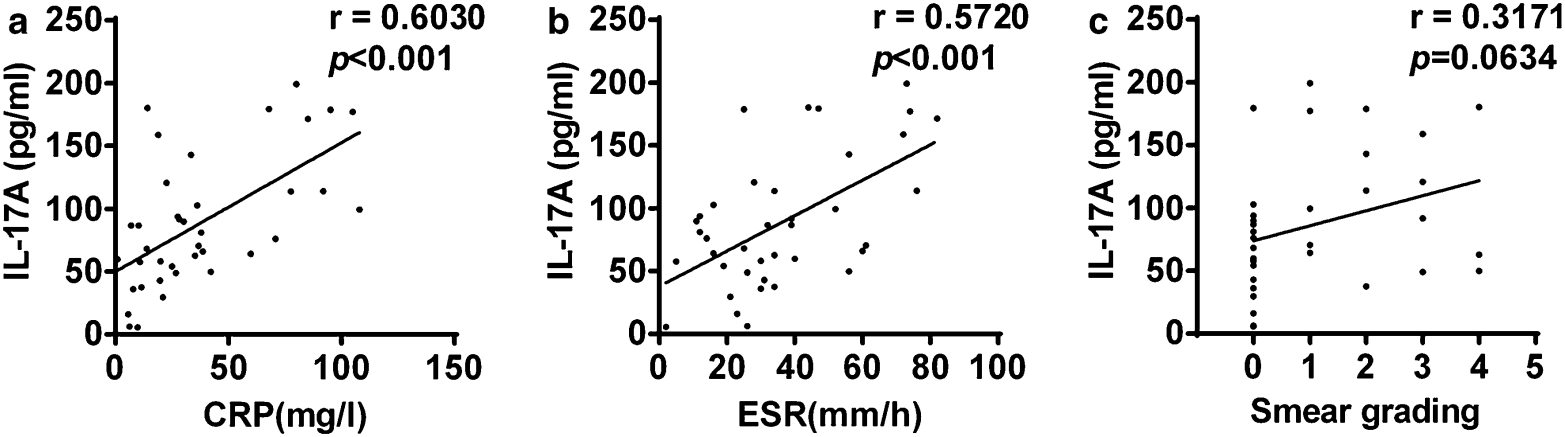
Correlation between the severity of the disease and the IL-17 level. **a** A Pearson’s correlation coefficient analysis between IL-17 and C-reactive protein (CRP) in patients was performed. **b** Pearson correlation coefficient analysis between IL-17 and erythrocyte sedimentation rate (ESR) in patients was performed

Previous studies had reported that CRP and ESR could be considered as severity indicators of pulmonary TB [23–26]. To investigate the relationship between IL-17 levels and the severity of pulmonary TB, the correlation between IL-17 level and CRP as well as ESR were conducted. We found that IL-17 level presented a significantly positive correlation with CRP level (r = 0.6030, p < 0.001) (Fig. 4a), and that IL-17 level was also positively correlated to ESR level (r = 0.5720, p < 0.001) (Fig. 4b). These data suggested that IL-17 level might have some correlation with the severity of pulmonary TB.

### IL-17 could aggravate alveolar epithelial cells damage after BCG stimulation in vitro

**Fig. 5:**
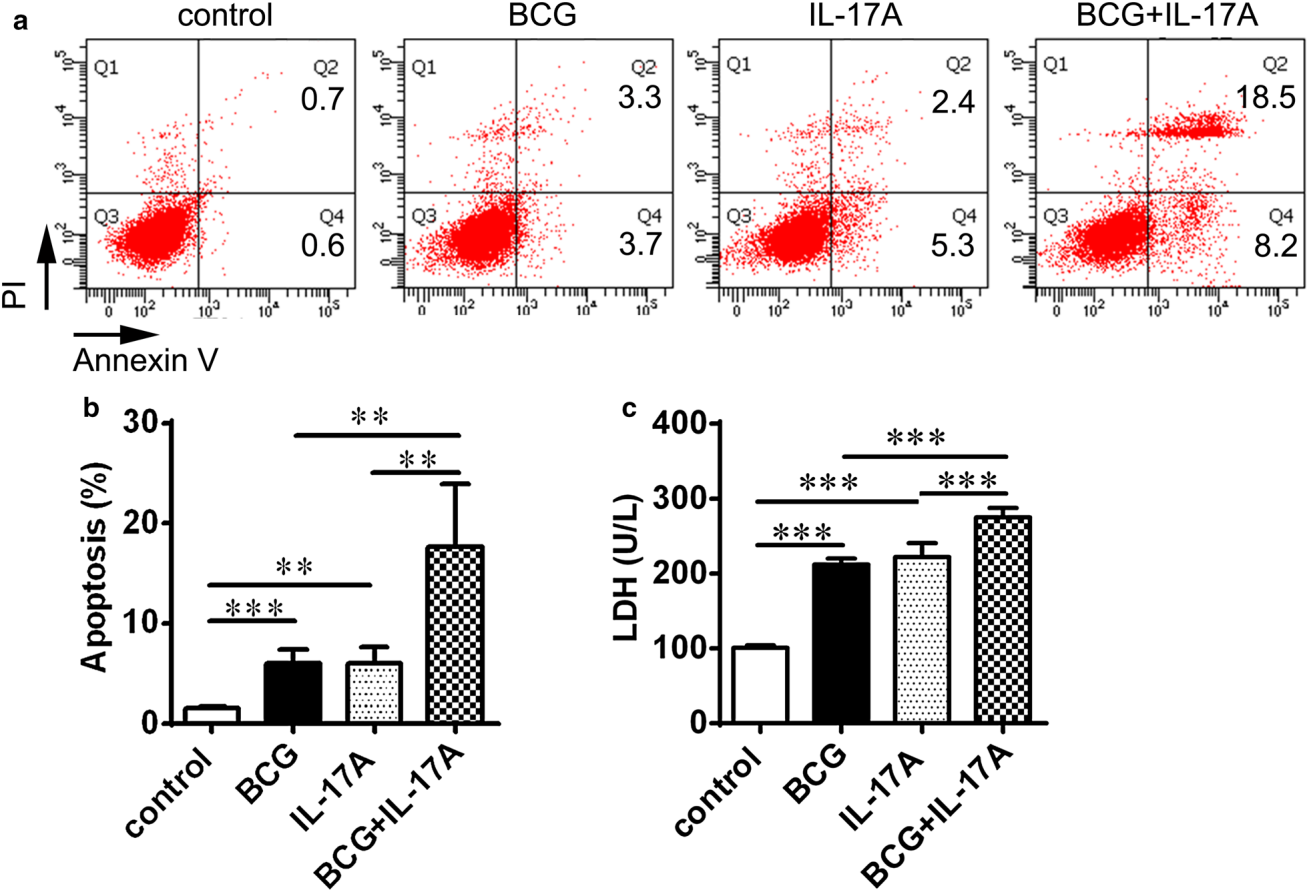
The effect of IL-17 on alveolar epithelial cells. **a** The *scatterplots* and **b** the percentage of apoptosis (early apoptosis Q2 and late apoptosis Q4) in alveolar epithelial cells 24 h after stimulation by IL-17 alone, or by IL-17 combined with BCG. **c** The levels of LDH released by alveolar epithelial cells 24 h after stimulation with IL-17 alone or combined with BCG. Data are presented as the mean ± SD. **p < 0.01; ***p < 0.001

Recent studies have shown that the IL-17 plays an important role in initial immune response and is involved in immune pathology in MTB infection [2, 9, 10]. Thus, we determined the influence of IL-17 on alveolar epithelial cells. We found that alveolar epithelial cells apoptosis was increased after IL-17 stimulation and BCG stimulation, respectively (Fig. 5a, b). Furthermore, IL-17 stimulation combined with BCG induced a higher level of cells apoptosis compared to IL-17 stimulation alone (Fig. 5a, b). These data indicated that IL-17 might induce alveolar epithelial cells apoptosis during MTB infection (Fig. 5a, b).

LDH is a cytosolic enzyme that is released upon cell damage [27], and could be released by alveolar epithelial cells. To further confirm the damage IL-17 on alveolar epithelial cells, we examined the level of LDH released by alveolar epithelial cells after IL-17 treatment in vitro. The level of LDH released from alveolar epithelial cells was increased after IL-17 treatment. IL-17 combined with BCG could enhance the release of LDH from alveolar epithelial cells (Fig. 5c). These results suggested that IL-17 could induce alveolar epithelial cells damage and aggravate lung injury in MTB infection.

### IL-17 secretion might be regulated by Treg cell and IL-10 after MTB infection

**Fig. 6:**
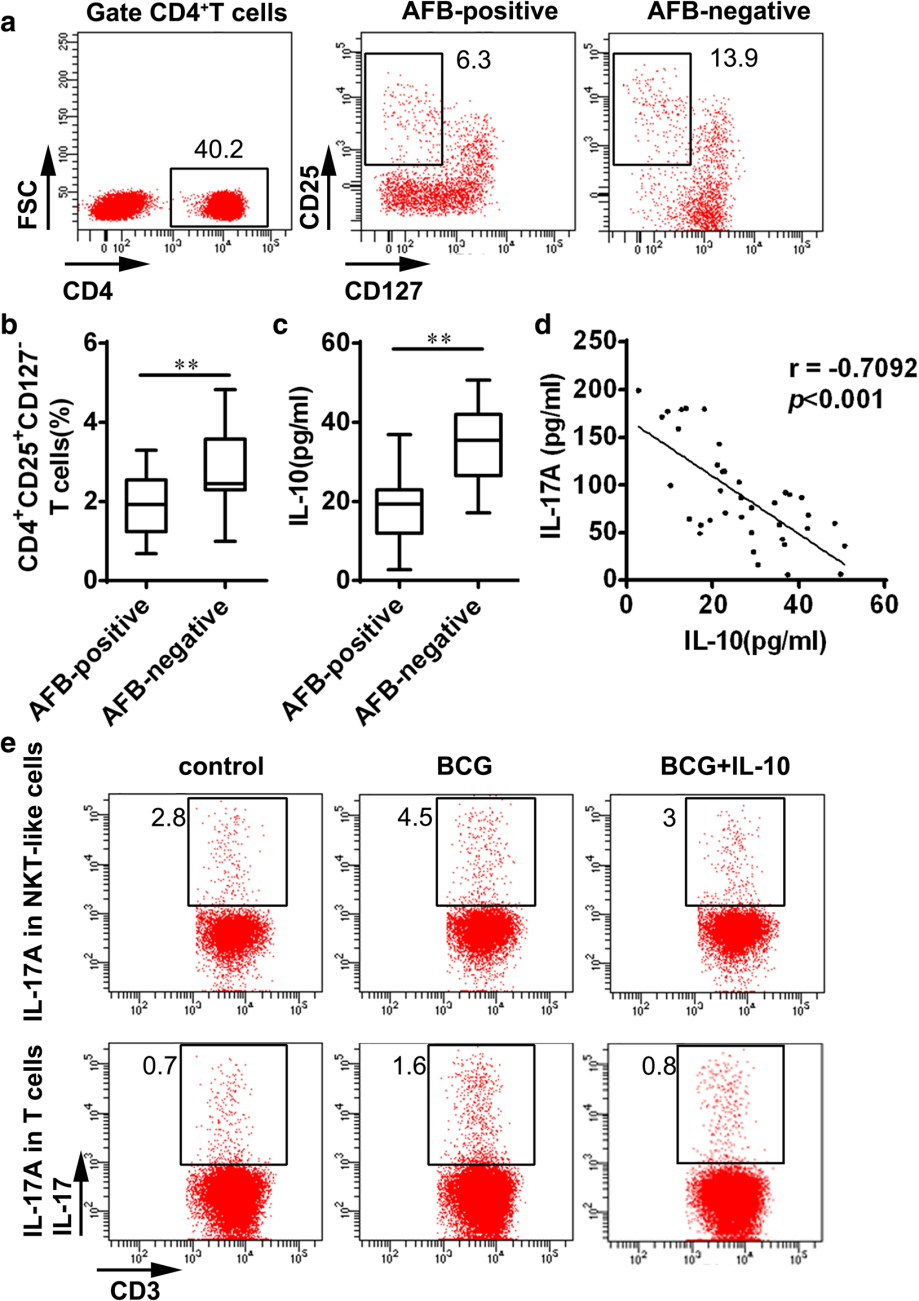
IL-17 secretion might be regulated by Treg cell and IL-10 in MTB infection. **a** The *scatterplots* and **b** the frequencies of Treg cells in PBMCs were presented. **c** Plasma levels of IL-10 in the AFB-positive group compared to AFB-negative group were analyzed by ELISA. Data were presented by *box plot*. **p < 0.01. **d** Correlation analysis between IL-10 and IL-17 in patients was analyzed. **e** The frequencies of IL-17-producing cell in NKT-like cells and T cells after stimulation with BCG alone or combined with rIL-10 were analyzed by flow cytometry

Recently, some studies have indicated that Treg cells may be an important protective factor in the progression of immune pathological damage by inhibiting Th17 cell response in a series of diseases including chronic hepatitis B [28, 29] and autoimmune disorders [30, 31]. Treg cells could produce amounts of IL-10 [32–34]. In MTB infection, Treg cells and IL-10 could also limit the immune pathological damage [19, 20, 33]. To investigate the potential mechanism of IL-17 alteration during anti-TB treatment, we observed Treg cells distribution and IL-10 expression. The percentage of Treg cells was remarkably elevated in the AFB-negative group compared to AFB-positive group (p = 0.0084) (Fig. 6a, b). Meanwhile, plasma IL-10 level was significantly increased in the AFB-negative group in comparison with AFB-positive group (p < 0.001) (Fig. 6c). Furthermore, we analyzed the correlation between IL-17 level and IL-10 level, and found that plasma IL-17 level had a significantly negative correlation with IL-10 level (r = −0.7092, p < 0.001) (Fig. 6d).

To further illustrate the function of IL-10 on IL-17 secretion during TB infection, we determined the frequency of IL-17-producing cells in healthy PBMCs after BCG stimulation combined with recombinant IL-10. We found that the percentages of IL-17-producing cells in NKT-like cells and IL-17-producing cells in T cells were increased after BCG stimulation (Fig. 6e). The administration of IL-10 could decrease the frequencies of IL-17-producing cells in NKT-like cells and IL-17-producing cells in T cells induced by BCG stimulation (Fig. 6e). These data indicated that Treg cells and IL-10 might inhibit the production of IL-17 during anti-TB treatment.

## Discussion

Tuberculosis is still a major public health problem in the world. Though a series of studies have shown that IFN-γ and CD4^+^ T cells play important roles in controlling both bacterial growth and immunopathology during MTB infection [2–4, 35, 36], the Th1 cells (primarily CD4^+^ cells producing IFN-γ) alone is not enough to control the infection [3, 5]. While previous studies have shown that IL-17 is associated with both protection and pathology in the context of an MTB infection [2, 9, 10], the changes in IL-17 production following anti-TB treatment remains unknown.

In this study, we found that plasma IL-17 level was increased in AFB-positive patients, but obviously decreased after smear conversion. IL-17 is a proinflammatory cytokine mainly produced by Th17 lymphocytes, which play an immunoregulatory role by producing a unique spectrum of pro-inflammatory cytokines IL-17A, IL-17F, IL-22, IL-26, and IFN-γ [11]. These cytokines could induce the activation and recruitment of neutrophils, macrophages, and Th1 lymphocytes into the site of infection, which contributed to delimitation of the damaged area in the lung tissue, as well as the inhibition of MTB growth [37]. In contrast, other studies demonstrated that IL-17 played an essential role in the formation of granuloma in the lung [17], and was involved in pathological damage in the lung by initial neutrophil recruitment after MTB infection [18]. In our study, we found that IL-17, especially combined BCG, could induce alveolar epithelial cells apoptosis and LDH release, which indicated that the increase of IL-17 in AFB-positive pulmonary TB patients might induce lung pathological damage.

T cells are considered as an important source of IL-17 [6, 7, 21]. NKT cells are distinct innate immune T cells which play a key role in the pathogenesis of various immunomediated liver diseases [38, 39]. In MTB infection, NKT cells could produce IL-21 to help B cells for the production of immunoglobulins [40] and participate in the local immune response against MTB through the production of IFN-γ and the secretion of cytolytic molecules [41]. CD3^+^CD56^+^ cells are not classical invariant NKT cells, but CD3^+^CD56^+^ cells are co-expressing T cell receptor and NK cell receptors. Recently, it was reported that activated NKT-like cells (CD3^+^CD56^+^) were capable of rapidly producing IL-17 [22, 42]. Our previous study showed that the percentage of NKT-like cells was significantly decreased after telbivudine therapy in chronic hepatitis B patients, and that a positive correlation between the frequency of NKT-like cells and serum HBV DNA level was observed [22]. In all pulmonary TB patients, irrespective of the clinical form (infiltrative pulmonary TB and disseminated pulmonary TB) and variant of the MTB infection (drug-sensitive pulmonary TB and drug-resistant pulmonary TB), the content of IL-17^+^ NKT-like cell in the peripheral blood was elevated compared to healthy individuals [11]. In our study, we found that the frequency of IL-17-producing cells in NKT-like cells was significantly decreased in the AFB-negative group in comparison with AFB-positive group. These data were consistent with the changes of plasma IL-17 level after anti-TB treatment, and suggested that NKT-like cells might also be the main source of IL-17, in addition to conventional T cells in TB patients.

Patients with AFB-positive pulmonary TB are highly infectious. Sputum smear conversion is that the growth of acid-fast bacilli in the lung is controlled, which is associated with a reduction in the rate of treatment failure and relapse [43, 44]. In our study, we found that plasma IL-17 level and the frequency of IL-17-producing cells in NKT-like cells were decreased after anti-TB treatment. Both CRP and ESR are considered as prognostic indicators in patients with pulmonary TB [24–26]. In our study, we found that the increase of IL-17 was correlated to both CRP and ESR, and could aggravate alveolar epithelial cells damage after BCG stimulation. These data indicated that the decrease of plasma IL-17 level and IL-17-producing cells in NKT-like cells frequency after anti-TB treatment might be beneficial for patients. However, the regulation of IL-17 alteration during treatment is still unclear.

Recently, some studies have indicated that Treg cells may be an important protective factor in the progression of immune pathological damage by inhibiting Th17 cell response in a series of diseases, such as chronic hepatitis B [28, 29] and autoimmune disorder [30, 31]. Treg cells could produce amounts of IL-10 [32–34]. In MTB infection, Treg cells and IL-10 also could limit the immune pathological damage [19, 20]. In our study, we found that the percentage of Treg cells was remarkably elevated in the AFB-negative group compared to the AFB-positive group. This was in opposition to the trend exhibited by the frequency of IL-17-producing cells in NKT-like cells. Moreover, the plasma IL-17 level had a significantly negative correlation with IL-10 level. Furthermore, IL-10 could decrease the percentages of IL-17-producing cells in NKT-like cells and IL-17-producing cells in T cells induced by BCG. These data indicated that the increase of the frequency of Tregs and IL-10 level following TB treatment could inhibit the expression of IL-17. This effect may function to alleviate the extent of immune injury in TB patients.

## Conclusions

In summary, we found that IL-17 was increased in AFB-positive pulmonary TB patients, but obviously substantially decreased after treatment following anti-TB treatment. Additionally, NKT-like cells might be an important source of IL-17, besides in addition to conventional T cells in TB patients. IL-17 could was also demonstrated to induce lung injury and epithelial apoptosis following MTB infection in MTB infection. The secretion of IL-17 may be regulated by Treg cells and IL-10 during anti-TB treatment. These results suggest that IL-17-producing NKT-like cells may play an important role in pulmonary TB patients.
